# Impact of palm kernel cake with or without multi-blend enzyme on the growth performance and carcass traits of Sasso broilers

**DOI:** 10.1080/23144599.2022.2125735

**Published:** 2022-09-29

**Authors:** A. A.-A. Koranteng, K. A. Gbogbo, B. Adjei-Mensah, T. Bouassi, C. T. F. Aïna, J. Glago, Tona Kokou

**Affiliations:** a Centre d’Excellence Régional Sur Les Sciences Aviaires, University of Lomé, Lomé, Togo; b Laboratoire de Botanique et Écologie Végétale, Faculté des Sciences, Université de Lomé, Lomé, Togo; c Togolese Institute of Agronomic Research, Kara, Togo

**Keywords:** Sasso broiler, palm kernel cake, multi-blend enzyme, non-conventional feed

## Abstract

Non-conventional feeds help alleviate competition faced by the poultry industry as the prices of conventional poultry feed ingredients, are continually increasing. Thus, the objective of this study was to evaluate the effect of palm kernel cake (PKC) on the performance of Sasso X44 broiler chicks. Four hundred and fifty (450) unsexed 21-day-old broiler chicks of homogenous weight were randomly allocated to five dietary treatments in a completely randomized design with six replicates having 15 birds per replicate. Diets comprised the control, PKC0 (basal broiler diet), PKC10 (10% PKC diet), PKC10 + E (10% PKC diet+ 0.05% enzyme), PKC20 (20% PKC diet) and PKC20 + E (20% PKC diet + 0.05% enzyme). Data were collected on feed intake, body weight gain, feed conversion ratio (FCR), and carcase parameters. Results revealed that birds in the PKC10 + E group obtained improved (p < 0.05) FCR compared to the control group at the grower phase. At the finisher phase, the PKC20 + E group consumed more feed (p < 0.05), which was significantly different from the other groups except for the PKC10 + E group. Average daily body weight gain was highest for birds fed PKC10 + E diet, which, was significantly different (p < 0.05) from birds fed PKC20 diet. The percent dressed weight was significantly superior [p < 0.05) for birds fed PKC10 + E and PKC10 relative to PKC20. Sasso broilers could therefore benefit from a diet partially replaced with 10% palm kernel cake incorporated with multi-blend enzyme.

## 1. Introduction

Poultry feed ingredients especially cereals have suffered fluctuations in prices. [1],has confirmed that the prices of conventional feed ingredients such as soybean meal and maize fluctuate with seasons and the level of production. The high cost of grains coupled with competition from humans [2] warrants poultry nutritionists to explore alternative feed sources (non-conventional agro-industrial by-products) for the feeding of poultry. Non-conventional feeds could partially bridge the gap in the feed supply, decrease competition for food between humans and animals [3], and ultimately help reduce the cost of production. Nevertheless, some of these non-conventional feeds are high in fibre and may contain some anti-nutritional factors (ANF) resulting in the inability of the birds to make efficient use of the ingested feed. Though broilers require fibre for the proper functioning of the digestive tract, their response to fibre inclusion depends on the source and level of dietary fibre, the characteristics of the diet, as well as the physiological status and health of the birds [4]. Broiler birds have, however, been fed on different sources of non-conventional feed ingredients such as corncob [5], palm kernel cake [6,7], rice husks [8] and cocoa pod husk [9,10].

Palm kernel cake (PKC), a solid by-product of oil palm (*Elaeis guineensis*) kernel oil extraction [11] has been extensively used to feed ruminants [12] and poultry [13]. PKC can be an alternative feed resource for poultry feed as humans do not use it as food [13]. It is highly nutritious, containing about 50.3% carbohydrate, 19.8% protein, 16.7% crude fibre, and 8% oil [14]. Notwithstanding the nutrient content of PKC, it also has anti-nutrients such as tannic acid, phytic acid, and oxalate [15], which are capable of interfering with the digestion process in broilers, therefore limiting its utilization [16]. Broilers have been fed up to a 30% inclusion rate of PKC during the finisher phase to yield comparable growth performance results with their 0% PKC counterparts [17]. [18] concluded that PKC could be included in the diet of Hubbard broiler chicks at 10% without any deleterious effect on growth performance and carcase traits but strongly recommended the supplementation of an enzyme at a 20% inclusion rate to improve the birds’ health and immune response. Sasso chicken is a dual-purpose bird that requires to be explored in terms of performance.

Since broilers have little or no endogenous enzymes and cellulolytic bacteria in their gastrointestinal tract required for non-starch polysaccharides and crude fibre digestion [19], there is the need to include exogenous enzymes to enhance feed efficiency and growth performance [19] when fed high fibre diet. Compared to other breeds of broilers, it takes a relatively long time for Sasso broilers to reach a marketable age and so necessitates alternative sources of cheaper but nutritious feed to enhance their performance and reduce their production cost. There is, however, a scarcity of information on the growth and carcase performance of Sasso broilers fed diets containing PKC. It was hypothesized that the supplementation of PKC incorporated with a multi-blend enzyme in the diet will improve the overall performance of Sasso broilers. Hence, the objective of this study was to evaluate the growth performance and carcase quality of Sasso broilers fed different levels of PKC with or without enzymes.

## 2. Materials and methods

### 2.1. Study area, birds husbandry and experimental design

The study was carried out at the experimental unit of the Centre d’Excellence Régional Sur Les Sciences Aviaires (CERSA) of the University of Lomé, Togo. Four hundred and fifty (450) unsexed 21-day-old dual-purpose broiler chicks [Sasso X44) obtained from Maison Diop de Lomé Hatchery, Lomé, Togo were randomly allocated to five dietary treatments having six replicates with 15 birds per replicate in a completely randomized design. The chicks were weighed and individuals with an average weight of 246 ± 6 g were selected and randomly placed in their respective treatment open-sided poultry pens. The experiment lasted for 9 weeks. The birds had access to feed and water *ad libitum* with 12 hours of light. They were raised on a floor littered with wood shavings of 4 cm thickness with a stocking density of 10 birds/m^2^. Vaccination of birds and prophylaxes were administered as recommended for Sasso breed.

### 2.2. Ethical statement

The study was conducted following the guidelines of the [20],and the recommendations of the Animal Ethics Committee of the University of Lomé provided animal care guidelines, which were keenly followed for this study with the reference number, ref:008/2021/BC-BPA/FDS/UL.

### 2.3. Experimental rations

Experimental feeds were formulated according to [21],with the specification of broiler feed requirements, which has been recommended for research on Sasso in the study institute. Diets were formulated to be both iso-caloric and iso-nitrogenous. The feed composition and proximate analysis results are presented in Table 1. Five experimental diets were formulated namely; PKC0 (Control diet, 0% PKC], PKC10 (10% PKC diet without multi-blend enzyme), PKC10 + E (10% PKC diet with 0.05% multi-blend enzyme), PKC20 (20% PKC diet without multi-blend enzyme), and PKC20 + E (20% PKC diet with 0.05% multi-blend enzyme). The multi-blend enzyme (Kemzyme Plus P) used is a commercial product from Kemin Industries, South Africa and the main active ingredients were xylanase, β-glucanase, cellulase, α-amylase, protease, and phytase. The PKC (a residue obtained from the extraction of oil for the production of palm kernel oil) was obtained from GVC Ave Palm, Assahoun, Togo and was milled before inclusion into the diets.

**Table 1.** Ingredients and nutritional composition of experimental diets.

INGREDIENTS (%)	PKC0	PKC10	PKC10 + E	PKC20	PKC20 + E
Maize	64.35	56.35	56.35	51.35	51.35
Fishmeal	2.00	2.00	2.00	2.00	2.00
Roasted soya	25.15	23.00	23.00	22.80	22.80
Wheat bran	5.00	5.00	5.00	-	-
PKC	-	10.00	10.00	20.00	20.00
Oyster shell	1.50	1.80	1.80	2.00	2.00
DCP	1.00	1.00	1.00	1.00	1.00
Vitamin Premix	0.30	0.30	0.30	0.30	0.30
Methionine	0.15	0.15	0.15	0.15	0.15
Lysine	0.20	0.20	0.20	0.20	0.20
Salt	0.35	0.20	0.20	0.20	0.20
Multi-blend Enzyme	-	-	0.05	-	0.05
Total	**100.00**	**100.00**	**100.00**	**100.00**	**100.00**
Nutrient analysis (%)					
Moisture content	9.54	10.37	10.33	10.97	10.04
Ash content	5.65	6.33	6.42	6.55	6.50
Crude protein	19.24	19.31	19.25	19.19	19.29
Crude fat	5.12	5.39	5.34	5.41	5.49
Crude fibre	3.98	5.15	5.12	5.19	5.21
Lysine	1.41	1.49	1.52	1.61	1.63
Methionine	0.64	0.66	0.71	0.81	0.83
NFE	50.73	51.25	46.28	49.31	48.85
Metabolizable energy (Kcal/kg)	3057	3077	3049	3053	3087

PKC = Palm kernel cake, DCP = Dicalcium phosphate

### 2.4. Chemical and proximate analysis

Analysis was done on the PKC for anti-nutrients (tannin, saponin, phytate, oxalate) and non-starch polysaccharides including cellulose, lignin, acid detergent fibre (ADF), and neutral detergent fibre [NDF). Proximate analysis was conducted on treatment diets with the [22] method. Tannins and saponins levels in the PKC were determined by the method described by [23]. Cellulose, ADF, and NDF were determined based on the procedure of [24]. The oxalate was determined by the method described by [25]. Phytate was determined by the method of [26].

### 2.5. Measurements and data collection

Daily feed intake (DFI] was recorded and birds were weighed individually on weekly basis for all replicates. Daily weight gain (DWG) and feed conversion ratio (FCR) were calculated during both grower (4–8 weeks old) and finisher phases (9–12 weeks old), though single feed was served during the entire period, based on the recommendation for Sasso breed at the study institution. This recommendation has been approved through a number of feeding trial studies conducted in the study area on the Sasso breed. At the end of the experimental period, two birds from each replicate were selected based on the average body weight of the group and humanely sacrificed by severing the jugular vein after being fasted (with free access to water) for 12 hours. The carcases were used for carcase analysis, internal organ weights and morphometric measurements. The carcases were de-feathered and eviscerated. The carcase was cut up into different portions [breast, back, thigh, drumstick, both wing, neck, and abdominal fat) according to the procedure described by [27],with some modifications made. The weights of the dressed carcase, carcase cuts and abdominal fat were expressed as a percentage of the fasted live weight as indicated below;

$$DWG=\frac{weekly\;\;weight}{7}$$

$$FCR=\frac{feed\;\;intake}{\;weight\;\;gain}$$

$$Cut-up\;\;part\;\left(\%\right)=\frac{\;\;weight\;of\;cut-up\;part}{fasted\;live\;weight}*100$$

$$Dressed\;\left(\;\%\right)=\frac{Dressed\;weight}{fasted\;live\;weight}*100$$

The liver, gizzard, heart, and spleen were carefully removed and the weight of each replicate was expressed as a percentage of the live weight.

$$Relative\;organ\;weight\;\left(\;\%\right)=\frac{Organ\;weight}{fasted\;live\;weight}*100$$

The small intestine was divided into its segments: duodenum (from gizzard to entry of the bile and pancreatic ducts], jejunum (from entry of the ducts to yolk stalk), and ileum (from yolk stalk to ileocaecal junction). The weights and the length of the small intestine segments, the caeca and the colon were recorded. The length was taken by vertically suspending the segment on a hook in order to facilitate the measurement using a tape metre.

### 2.6. Statistical analysis of data

Data obtained on feed intake, body weight, and carcase parameters were subjected to a one-way analysis of variance (**ANOVA**) in a completely randomized design using the procedure of Minitab (Version 19), at a 5% level of significance and significantly different means were tested using Tukey’s pairwise comparisons of the same statistical software.

## 3. Results

### 3.1. Chemical composition/analytical evaluation of PKC

The anti-nutritional factors (tannin, saponin, phytate, and oxalate) and the fibre fractions (ADF, NDF, hemicellulose, cellulose, and lignin) of raw PKC is presented in Table 2. The ADF and NDF were, respectively, 35.62% and 60.20%. The lignin was 7.75%, but hemicellulose (24.58%) and cellulose (24.31%) were almost the same. The highest anti-nutrient in the PKC was tannin (3500 ppm), whilst the lowest was oxalate (13.4 ppm). The values of saponin and phytate were 690 and 109.50 ppm respectively.

**Table 2.** Fibre fractions and Anti-nutritional factors of palm kernel cake.

Parameter	Composition
Acid Detergent fibre (%)	35.62
Neutral Detergent fibre (%)	60.20
Hemicellulose (%)	24.58
Cellulose (%)	24.31
Lignin (%)	7.75
Tannin (ppm)	3500
Saponin (ppm)	690
Phytate (ppm)	109.50
Oxalate (ppm)	13.4

### 3.2. Growth performance

The effect of supplementation of broiler diet with PKC with or without multi-blend enzyme on the growth performance of Sasso broiler is shown in Table 3. At the end of the growing phase, birds fed the PKC10 + E diet had significantly (p < 0.05) lower DFI when compared to the other treatments except for the PKC10 group. The birds fed the control diet had the highest feed consumption. The DFI of the control group was not significantly different from birds fed the PKC20 + E diet. No significant differences (p > 0.05) were observed across all the treatment groups in ABW and DWG. An improved FCR was recorded for the birds fed the PKC10 + E which was significantly higher than (p < 0.05) that of birds fed the control diet.

**Table 3.** Effect of palm kernel cake with or without enzymes diet on the growth performance of Sasso broilers.

		TREATMENTS						
	PARAMETER	PKC0	PKC10	PKC10+E	PKC20	PKC20+E	SEM	P-value
	IBW	242.20	246.85	245.80	250.81	246.63	11.20	0.968
	DFI (g)	71.22^a^	67.39^bc^	66.25^c^	68.95^b^	71.30^a^	0.76	0.000
	ABW (g)	937.40	946.60	973.46	932.20	961.80	29.70	0.622
	DWG (g)	19.85	19.97	20.79	19.49	20.45	0.69	0.383
GROWER PHASE	FCR	3.62^a^	3.38^ab^	3.19^b^	3.54^ab^	3.50^ab^	0.13	0.033
FINISHER PHASE								
	IBW (g)	937.40	946.60	973.46	932.20	961.80	29.70	0.622
	DFI (g)	92.35^b^	89.25^b^	95.93^ab^	93.93^b^	102.78^a^	2.44	0.000
	ABW (g)	1733.40 ^ab^	1759.90^ab^	1812.30^a^	1692.70^b^	1782.30^ab^	33.20	0.019
	DWG (g)	29.33^ab^	27.867^ab^	30.61^a^	25.79^b^	28.54^ab^	1.22	0.011
	FCR	3.17 ^b^	3.21 ^b^	3.14 ^b^	3.65 ^a^	3.60 ^a^	0.13	0.001

^abc^*Means with different superscripts within a row are significantly different from each other at P < 0.05. SEM = Standard error of mean, P- value = probability value*

*PKC0 (basal broiler diet), PKC10 (10% palm kernel cake diet without multi-blend enzyme), PKC10 + E (10% palm kernel cake diet with multi-blend enzyme), PKC20 (20% palm kernel cake diet without multi-blend enzyme) and PKC20 + E (20% palm kernel cake diet with multi-blend enzyme)*

*IBW = Initial body weight*

*DFI = Average daily feed intake*

*ABW = Average body weight*

*DWG = Average daily weight gain*

*FCR = Feed conversion ratio*

At the end of the finisher period, the PKC20 + E group consumed significantly (p < 0.05) higher quantities of feed than the other treatment groups except for the PKC10 + E treatment group. The initial body weight (IBW) at the beginning of the finisher phase was similar across all the treatments. The average body weight was highest for birds fed the PKC10 + E diet, which, was significantly (p < 0.05) higher than those of the birds fed the PKC20 diet. DWG of birds fed the PKC10 + E diet was higher (p < 0.05) than the PKC20 group but similar (p > 0.05) to the Control and PKC20 + E groups. Birds fed the control, PKC10 and PKC10 + E diet had similar (p > 0.05) FCR and these in turn were superior to those fed diets containing PKC20 and PKC20 + E.

### 3.3. Carcase characteristics

The effect of supplementation of PKC with or without multi-blend enzymes on Sasso broilers is presented in Table 4. Birds fed the enzyme supplemented diets (PKC10 + E and PKC20 + E) had fasted live weights similar to the control and the PKC10 but higher than those fed diets containing PKC20. The percent dressed weight of birds fed the PKC10 + E and PKC10 were similar (p > 0.05) to the control but PKC20. Birds fed PKC10 + E diets recorded lower percent shank weight but had a significantly higher (p < 0.05) percent abdominal fat than all the other treatment groups. The inclusion of the diet with PKC with or without multi-blend enzyme did not affect the percent neck weight of the birds.

**Table 4.** Effect of addition of palm kernel cake with or without enzymes on the carcase characteristics of Sasso broilers.

PARAMETER	TREATMENTS						
	PKC0	PKC10	PKC10 + E	PKC20	PKC20 + E	SEM	P-value
Fasted live weight	1649.90^ab^	1679.80^ab^	1741.20^a^	1592.20^b^	1695.20^a^	33.90	0.004
% Dressed weight	76.87^ab^	77.32^a^	77.03^a^	76.17^b^	76.18^b^	0.40	0.031
% Neck weight	6.13	5.89	5.95	5.95	5.90	0.23	0.836
% Shank weight	4.34^a^	4.47^a^	3.26^b^	4.08^a^	4.24^a^	0.26	0.001
% Abd fat weight	1.50^b^	1.25^b^	2.37^a^	0.70^b^	1.03^b^	0.28	0.000
% Wing weight	9.43^ab^	9.63^a^	8.70^c^	9.61^a^	9.11^b^	0.14	0.000
% Drumstick weight	10.87^a^	10.46^ab^	9.56^b^	10.33^ab^	10.30^ab^	0.34	0.016
% Thigh weight	10.00	9.43	9.33	9.58	9.23	0.33	0.182
% Back weight	15.85^ab^	16.13^ab^	16.96^a^	15.34^b^	15.58^b^	0.45	0.017
% Breast weight	18.28^b^	19.28^ab^	20.80^a^	18.55^b^	19.07^ab^	0.60	0.004

^abc^*Means with different superscripts within a row are significantly different from each other at P < 0.05. SEM = Standard error of mean, P- value = probability value*

*PKC0 (basal broiler diet), PKC10 (10% palm kernel cake diet), PKC10 + E (10% palm kernel cake diet with multi-blend enzyme), PKC20 (20% palm kernel cake diet) and PKC20 + E (20% palm kernel cake diet with multi-blend enzyme)*

*Abd = abdominal*

The percent wing weight of birds fed the PKC10 + E diet was lower (p < 0.05) from birds in all the other treatment groups (Table 4). Birds fed the control diet recorded had the highest percent drumstick weight, which was significantly higher (p < 0.05) than that recorded for birds fed the PKC10 + E diet. Birds fed the PKC10 + E diets recorded significantly higher percent back weight (p < 0.05) relative to those fed the PKC20 and PKC20 + E diets. Percent thigh weight was not affected (p > 0.05) by PKC and enzyme supplementation in the diet of the birds.

### 3.4. Internal Organ weights and morphometry

The results for the effect of supplementation of PKC incorporated with or without multi-blend enzyme on the internal organ weights of Sasso broilers are shown in Table 5. The weight of full GIT for birds fed PKC20 and PKC20 + E diets were significantly higher (p < 0.05) than that of birds fed the PKC10 + E diet. The weights of full and empty gizzard were highest (p < 0.05) for birds fed the PKC20 and PKC20 + E diets compared to the other treatments diets. However, the incorporation of PKC and enzyme in the diets of the birds did not influence the heart, liver and spleen weights. Results for the morphometric analysis of Sasso broilers fed the palm kernel cake with or without enzymes diets are shown in Table 6. The small intestine of broilers fed PKC10 + E was lower than (p < 0.05) those fed PKC20 but was similar to the other treatments (p > 0.05). The duodenal length was longer (p < 0.05) for birds fed the PKC20 diet than that of the birds fed the control diet. The length of the jejunum was shortest for birds fed the PKC10 + E diet and this was significantly lower (p < 0.05) than those from PKC10 and PKC20 + E groups. The colon length was highest for birds fed the PKC20 + E diet, which was significantly lower (p < 0.05) than that from birds fed the PKC20 and PKC10 + E diets. The lengths of ileum and caecum were conversely not significantly (p > 0.05) affected by the addition of PKC and enzyme.

**Table 5.** Internal organ weights of Sasso broilers fed diets supplemented with palm kernel cake with or without enzymes.

PARAMETER (%)	TREATMENTS						
	PKC0	PKC10	PKC10 + E	PKC20	PKC20 + E	SEM	P-value
Full GIT	10.19^ab^	9.46^ab^	8.77^b^	11.02^a^	10.62^a^	0.55	0.004
Full gizzard	2.60^b^	2.88^b^	3.03^b^	3.70^a^	3.66^a^	0.17	0.000
Empty gizzard	1.20^b^	2.03^b^	2.20^b^	2.62^a^	2.68^a^	0.12	0.000
Heart	0.38	0.36	0.41	0.43	0.43	0.025	0.060
Liver	1.88	1.70	1.68	1.69	1.71	0.086	0.146
Spleen	0.15	0.15	0.16	0.18	0.21	0.02	0.063

^ab^*Means with different superscripts within a row are significantly different from each other at P < 0.05.*

*SEM = Standard error of mean, P- value = probability value*

*PKC0 (basal broiler diet PKC10 (10% palm kernel cake diet), PKC10 + E (10% palm kernel cake diet with multi-blend enzyme), PKC20 (20% palm kernel cake diet) and PKC20 + E (20% palm kernel cake diet with multi-blend enzyme)*

*GIT = gastrointestinal tract*

**Table 6.** Morphometric analysis of Sasso broilers fed palm kernel cake with or without enzymes diets.

PARAMETER (cm)	TREATMENTS						
	PKC0	PKC10	PKC10 + E	PKC20	PKC20 + E	SEM	P-value
Small intestine	160.91^ab^	160.88^ab^	143.24^b^	173.87^a^	167.35^ab^	9.70	0.028
Duodenum	25.54^b^	28.75^ab^	27.78^ab^	33.21^a^	30.10^ab^	2.14	0.024
Jejunum	31.68^bc^	44.97^a^	30.42^c^	41.20^abc^	42.40^ab^	3.71	0.002
Ileum	103.69	87.16	85.04	99.45	94.85	8.14	0.150
Caecum	18.000	18.70	16.05	18.20	18.91	1.44	0.324
Colon	8.39^ab^	8.20^ab^	7.70^b^	7.52^b^	9.70^a^	0.54	0.006

^abc^*Means with different superscripts within a row are significantly different from each other at P < 0.05. SEM = Standard error of mean, P- value = probability value*

*PKC0 (basal broiler diet), PKC10 (10% palm kernel cake diet), PKC10 + E (10% palm kernel cake diet with multi-blend enzyme), PKC20 (20% palm kernel cake diet) and PKC20 + E (20% palm kernel cake diet with multi-blend enzyme)*

## 4. Discussion

This study investigated the growth performance and carcase quality of Sasso broilers fed different levels of PKC supplemented with or without multi-blend enzymes. Several investigations conducted using PKC at different graded levels with some enzyme supplementation have yielded a positive impact on broilers [18,28,29]. Chemical analysis showed that PKC used in this study contains several levels of anti-nutritive factors and non-starch polysaccharides such as tannins, saponins, ADF and cellulose. The percent saponin and tannin present in the tested PKC were, respectively, 690 and 3500 ppm, which were higher than 260 ppm saponin and 310 ppm tannin reported by [30]. Anti-nutritional factors are substances found in most food substances responsible for deleterious effects, which limit their nutrient and micronutrient absorption [31]. Some of them such as oxalate interact with the nutrients in the gastrointestinal tract while tannins reduce the digestibility of dry matter, protein and other nutrients [32]. Results revealed that the oxalate and phytate levels present in the PKC were outside the lethal dose of 2–5 g/kg and 50–60 mg/kg, respectively, as reported by [33].

The ADF and NDF, hemicellulose, and cellulose values were similar to those of [34], who reported 36.14% – ADF, 61.54% – NDF, and 25.40% – hemicellulose but lower than [51.48% ADF, 82.29% NDF, 30.81% hemicellulose and 35.55% cellulose reported by [35]. The lignin content obtained from PKC for this study was lower than reported by [36]. According to [37], low levels of lignin promotes enzymatic hydrolysis as they can absorb enzymes thus preventing irreversible loss of enzyme activity. The differences in the values obtained for anti-nutrients and fibre composition could be attributed to the differences in geographical locations and species of the palm tree in addition to quality and the extraction and processing method used [11, 38].

At the end of the grower phase, the PKC10 and PKC20 (without enzymes) recorded a significantly (p < 0.05) reduced feed intake when compared to the control diet. This could be due to the bulkiness of the PKC as compared to maize, the possibility of the fibre impeding digestibility of the diet [29], and the probable unpalatable nature of the diet [18]. Our result partly aligns with the observations made by [18],and [27], who reported a relatively lower feed intake in birds fed with a 20% PKC diet, in contrast to those fed 10% PKC and the control diet. No significant differences were however observed by [6],when broilers were fed 0 and 10% PKC levels. The disparities between our result and those of other authors could be due to differences in the strain of birds used and their ability to handle different PKC inclusion levels without enzymes.

In this study, a significantly lower (p < 0.05) feed intake was recorded for birds fed the PKC10 + E diet compared to the birds fed the PKC20 + E and the control diets control, but similar (p > 0.05) to birds fed the PKC10 diet. The multi-blend enzyme incorporation in PKC10 + E might have enhanced nutrient digestibility due to the breakdown of non-starch polysaccharides by the activity of the enzymes [29], thereby reducing the need to consume more in order to meet body requirements. Exogenous enzyme supplementation enhances the digestibility of fibrous agricultural products [39]. Though the enzyme was incorporated in diet PKC20 + E, the 0.05% inclusion level may not have been adequate for the 20% level of PKC, thereby compelling the birds to consume more feed compared to the PKC10 + E group. [18], and [28], have reported an increased feed consumption with increasing PKC levels but when the enzyme was incorporated into the diet, it caused a decreased feed intake. The source and composition of the exogenous enzymes used for the various studies may have also contributed to the variation in results obtained.

At the end of the finisher phase, the effect of PKC20 + E on feed intake remained significantly higher than that of PKC10 and PKC20 as seen in the grower phase. Interestingly, birds fed the control diet had a significantly lower feed intake at the finisher phase in contrast to PKC20 + E, unlike the grower phase where they had similar feed intake. Feed intake level was similar in control, 10% and 20% PKC without enzyme. [7] also found no significant differences in feed intake between 0%, 10%, and 20% PKC levels of non-enzyme supplemented diets at the finisher phase of their experiment. In contrast, the result of [27] showed that at the finisher stage, 20% PKC inclusion significantly lowered feed intake compared to that of 0% and 10% PKC counterparts. [40] showed that a 20% PKC without enzyme recorded a significantly higher feed intake compared to 0% and 10% PKC levels, but when the 20% PKC was incorporated with Maxigrain enzymes, the bird consumed significantly more feed than the 10% PKC and 20% PKC diets. Also, [41] reported a higher feed intake for broilers fed 30 and 45% Purafex [a premium grade PKC-containing diet) compared to their control counterparts and pointed out that broilers will ingest more feed with these levels to attain their targeted body weight.

No significant differences were observed among all treatments in terms of ABW and DWG at the end of the grower period but at the end of the finisher phase, the ABW and DWG of the PKC10 + E group showed significantly higher values compared to the PKC20 group. Thus, it is beneficial to incorporate multi-blend enzymes in a 10% PKC inclusion level to achieve better body weight and weight gain rather than feeding a 20% PKC diet without enzymes. [40] found out at the end of 4 weeks that broilers fed 10% PKC incorporated with enzymes obtained a significantly higher ABW and DWG compared with their control, 10% PKC, 20% PKC, and 20% PKC+E groups. The authors observed also that the 10% PKC with enzymes supplemented group attained a DWG significantly higher than the control, 10% PKC and 20% PKC but similar to 20% PKC incorporated with enzyme group.

At the end of the growing period, the PKC10 + E fed birds were more efficient in feed conversion compared with the birds fed the control diet. This study agrees with [40], who established that 10% PKC infused with enzymes caused an improved FCR compared to the control and all the other treatments at the grower phase.

However, at the finisher phase, birds of the control group, PKC10, and PKC10 + E recorded a better FCR compared to the PKC20 and PKC20 + E groups. In agreement with our findings, [40] reported that at the finisher stage, birds fed the 10% PKC incorporated with enzymes again had improved FCR, which was better than only the 10% and 20% PKC diet. [11],is of the view that, palm kernel meal supplemented with enzymes contribute to greater nutrient digestibility as compared to enzyme-free diets, which result in a better FCR. Our result suggests that at the grower phase, PKC dietary inclusion at 10% supplemented with enzyme enhances feed conversion, whilst dietary inclusion of PKC at 20% with or without enzyme is detrimental to FCR. In another study, no effect was observed in the overall growth performance when broilers were fed with fermented PKC (20%] at the finisher phase [42]. Unpublished data (Koranteng) showed that the diets similar in composition with PKC20 and PKC20 + E had poor faecal crude fibre digestibility though there was no statistical difference, multi-blend enzyme inclusion in the 10% PKC diet yielded a better FCR [2.2%) over the PKC10 group. [18] found a 9.6% reduction between the 10% PKC and the 10% PKC enzyme-treated group although without any significant difference.

Multi-blend enzyme incorporated in PKC diet has a positive influence on the fasted live weight of Sasso broilers. In this study, birds fed PKC diet incorporated with multi-blend enzymes produced birds that were heavier than the non-enzyme supplemented group. This observation confirms the claim of [43] that, enzyme supplementation can improve the performance and nutrient digestibility of broilers fed with diets containing high levels of grains rich in non-starch polysaccharides. It is obvious from the results of this study that enzyme incorporation is required when the level of PKC is increased to 20%, as birds fed the PKC20 diet had rather the lowest live weight.

There was a clear distinction in dressed weight between PKC at 10% and 20% diet incorporation levels regardless of the inclusion or non-inclusion of the enzyme. To attain an appreciable percent dressed weight, the PKC level should be maintained at 10% because increasing the level to 20% caused a relative decline in weight. This agrees with [27], who observed a decreasing order of body weight and dressing percentage as PKC levels increased from 10% to 20%. In the case of 7, as the PKC level increased, the live weight and the percent dressed weight decreased even though not statistically, as observed in this study. On the other hand, [17], and [44] reported that palm kernel meal does not influence the dressing percentage of broilers. These contradictory findings could be attributed to the effect of the PKC extraction method used by different authors as reported by [38], and [11].

Nevertheless, neither PKC addition nor enzyme inclusion influenced the percent neck weight of Sasso broilers. [29] on the hand demonstrated that birds fed the control diet obtained a significantly high-percent neck weight compared to 10% and 20% PKC. Relatively low-percent shank weight was recorded in PKC10 + E fed birds compared to the other treatments. When considering PKC dietary inclusion levels, [17], and [7] documented no differences in percent weights of shanks when birds were given varying levels of PKC. In contrast to our result, [29] reported comparable leg weights of birds fed the control diet and 10% PKC (without enzyme] but were significantly superior to those fed 20% PKC [without enzyme). It is worthy of note that [29] demonstrated that enzyme incorporation increases the leg weight of broilers in contrast to the control. The differences between our findings and that of [29],may be due to the type of enzyme and its incorporation level. A reduction in shank weight caused by PKC10 + E in our study is of no economic implication.

The weight of the abdominal fat was significantly higher in PKC10 + E compared to the other treatments. This increased fat percentage might imply a higher rate of fat deposition. The result obtained in this study negates the findings of [29], who demonstrated that birds fed the control diet had the highest weight of abdominal fat in comparison to 10% and 20% PKC diets. Findings of [45] also indicated that the abdominal fat of birds fed palm kernel meal-containing diets was significantly lower than the control group. The disparity between our results and other studies might be attributed to the probable differences in the fat level of PKC used due to the extraction method. Interestingly, [29] highlighted that enzymatic action reduces fat deposition by breaking down nutrients for better utilization. The result of our study shows that the effect of PKC and enzyme inclusion on fat deposition needs to be further explored.

PKC10 + E treatment birds had significantly lower wing weight compared to all the other treatments and enzyme incorporation also further lowered the percent wing weight for both the 10% and 20% PKC levels. On the contrary, [29] demonstrated that there was no significant difference between the control and enzyme incorporated diets. The percent wing weight obtained by the control, PKC10, and PKC20 were not significantly different from each other. In the same light, results obtained by [27], and [29] showed no significant differences between the control, 10% PKC and 20% PKC. Similar percent drumstick weights were obtained for the control, PKC10, PKC20, and PKC20 + E groups whilst the percent drumstick weight was significantly higher for the birds fed the control diet than for birds fed the PKC10 + E diet. [29] has reported that birds fed an enzyme-incorporated diet had a superior drumstick weight compared to the non-enzyme group.

The percent back weight of the birds fed the PKC10 + E was significantly higher than those fed PKC20 and PKC20 + E diets. [29] reported no significant differences between control, PKC 10 and PKC 20. However, the authors reported an increased back weight when the enzyme was included.

Birds fed the PKC10 + E diet produced significantly high-percent breast weight compared to the control and PKC20 diets. The relatively heavy weights of the breast and back attained for the PKC10 + E group were at the expense of their wings and shanks. This is advantageous because the breast carries more economic value compared to the other parts. The PKC10 + E diet may have provided a steady intake of nutrients especially lysine and methionine that induces the synthesis of the breast relative to other muscles [46]. In contrast to our study, [29] reported a decrease in breast weight with an increase in PKC inclusion level with the control group having a higher weight, and the incorporation of the enzyme did not influence the breast weight. The discrepancies in our studies might be attributed to differences in the ration type, nutrient content, environmental conditions, management conditions, processing, and the strain of birds used, .

A recent study by [47] indicated that the size of the GIT increases as a result of the adaptation required to handle the introduction of dietary fibre. In this study, the relative weight of the full GIT of birds fed 20% PKC incorporated with multi-blend enzyme were heavier than those fed the PKC10 + E diet, indicating that dietary fibre might have been more in the PKC20 + E diet. Additionally, according to [48], there is evidence that the differences in the weight of organs are highly related to differences in the type of fibre. In this study, the incorporation of PKC and enzyme supplementation in the diets of the birds showed no differences in the heart, liver and spleen weights. In agreement, [49] did not observe any significant differences in liver weights of broilers fed diets containing 0, 5, 10, 15, and 20% PKC levels, whereas [29] found out that the liver weight of birds given the control diet had significantly heavier weights than those fed the 10% PKC but heart weight of the control group was lower than those fed the 10, 20 and 30% levels.

Gizzard weight increased with increasing levels of PKC in this study. Similarly, [17] found out that the addition of 15% PKC in the diet of birds resulted in a significantly higher percent empty gizzard weight than the control group. [29] are of the view that PKC is bulkier than maize and can thus cause a weight increase in the full gizzard as larger particles are selectively retained in the gizzard [50], which could contribute to the development of the gizzard muscles. [51] has also stipulated that gizzard has a quick response to changes in the coarseness of feed and this aids digestion by reducing the particle size and chemical degradation of nutrients in addition to the regulation of feed flow. Another school of thought indicates that there can be a substantial increase in the gizzard structural components when introduced with, e.g. hulls and larger cereal particles [51,52]. [53] also observed a rapid increase in the size of quail gizzard after being introduced high-fibre diet for 14 days. The author inferred that the consequent size increase was because of the stimulative effect of the grinding activity on the gizzard muscle.

The inclusion of PKC and enzyme supplementation did not influence spleen size in this study. This finding is supported by [6],had no significant differences in the relative weights of the spleen among groups of broilers fed graded levels of PKC. On the contrary, [29], and [54], who observed that diet supplemented with 0.1% enzyme cocktail significantly improved the weight of the spleen compared to their control group. It has been reported by [47] that dietary fibre could affect both the length and weight of the intestines. It was observed in this study that the length of the small intestine was the longest for birds fed 20% PKC. In addition, this same group of birds had the longest duodenal length, which may have contributed to the length of the intestine. [55] has also reported that the duodenal and ileum length was longer at 60 g/kg crude fibre than the 30 g/kg diets given to 30-day-old turkeys.

## 5. Conclusion

Dietary inclusion of PKC10 + E appears to be more beneficial to growth performance and carcase yield over the other treatments as it improves daily weight gain and breast muscle. It is however recommended that further research should be done to ascertain an inclusion level of the multi-blend enzyme for the 20% PKC inclusion, and the effect of PKC and enzyme inclusion on fat deposition needs to be further explored.
